# Optical, Chemical, and Biological Detection Methods of Microplastics and Nanoplastics

**DOI:** 10.1021/acsmeasuresciau.6c00028

**Published:** 2026-04-03

**Authors:** Carlos Vico, Song Lin Chua

**Affiliations:** † Department of Applied Biology and Chemical Technology, The Hong Kong Polytechnic University, Kowloon, Hong Kong SAR 999077, China; ‡ Research Centre for Deep Space Explorations (RCDSE), The Hong Kong Polytechnic University, Kowloon, Hong Kong SAR 999077, China; § Research Institute for Future Food (RiFood), The Hong Kong Polytechnic University, Kowloon, Hong Kong SAR 999077, China

**Keywords:** microplastics, nanoplastics, pollution, microscopy, spectroscopy, biosensors, mass spectrometry, environmental monitoring

## Abstract

Microplastics and nanoplastics (MNPs) are pervasive environmental contaminants with major impacts on ecosystems and human health. This raises the need to accurately detect and characterize MNPs to understand their sources, transport, fate, and biological effects. However, MNP sensing remains challenging due to their small size, chemical diversity, and complexity of environmental matrices. Here, we review recent advances in optical, chemical, and biological detection methods for MNPs as of Jan 2026 while comparing their strengths and limitations. Optical approaches, such as light microscopy, electron microscopy, and light scattering techniques, provide insights into size and morphology. Next, chemical detection, including FTIR, Raman spectroscopy, and mass spectrometry, enables quantitative and qualitative assessments of polymer types. Lastly, emerging biological strategies employ microbial biosensors and biomolecular probes for cost-effective, rapid, and in situ MNP detection. We also explore novel MNP-sensing methods and propose future directions for integrated and standardized MNP detection frameworks to support environmental monitoring and risk assessment.

## Graphical abstract

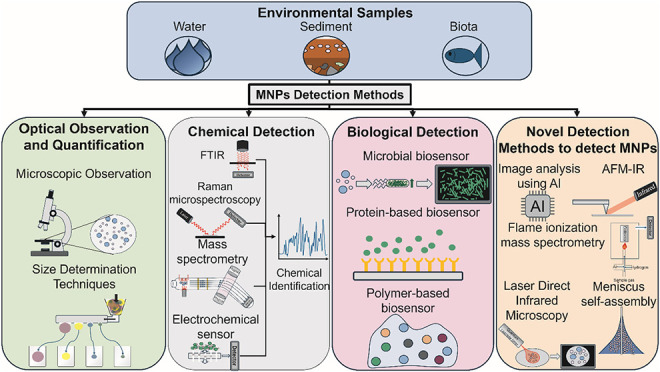

## 1 Introduction

Plastics are prevalently used in our lives, but the accumulation of plastic waste in the environment has raised major concerns. As of 2023, 450 million tons of plastic was produced, and around 350 million tons of plastic waste was produced each year.^1^ However, the danger of plastic pollution extends beyond large debris to include microplastics and nanoplastics (MNPs). Microplastics (MPs) are small plastics ranging from 100 nm to 5 mm, whereas nanoplastics (NPs) refer to plastics that are less than 100 nm in size.^2^ The accumulation of MNP waste causes adverse effects to both aquatic and terrestrial environments, and human health in the long run. MNPs can be easily ingested by organisms that mistake them for a source of food, which may lead to changes in their feeding behavior.^3–5^ Concern about MNPs’ exposure to humans has increased due to the increase in MNPs in the environment. The World Health Organization (WHO) emphasizes the need for further research on the toxic effects and long-term risks of MNPs in the human body.^6^ There are three main pathways through which MNPs could enter the human body, including ingestion of various consumables, secondary inhalation of MNPs in the air, and dermal absorption of MNPs through the skin via cosmetics and surgical or prosthetic devices.^7^ Consumption of MNPs could lead to organ damage, obstruction of the digestive tract, shock, debilitation, and, ultimately, death.^8^ The accumulation of MNPs in the human body can cause significant health risks.

The increasing concern about MNP pollution has raised the imperative need for the analysis and identification of MNPs to assess the extent of pollution (Figure 1). There are four steps to the identification and quantification of MNPs: sampling, separation, identification, and quantification (Figure 2). The primary regions that may be utilized to gather MNP samples are soil, water, associated sediment, and biota. Environmental MNP analysis involves collecting sediment, water, and biota samples using appropriate sampling methods, followed by density-based separation (also known as flotation), sieving/filtration, and chemical digestion-based purification to isolate MNPs for subsequent identification and quantification.^9^

**Figure 1:**
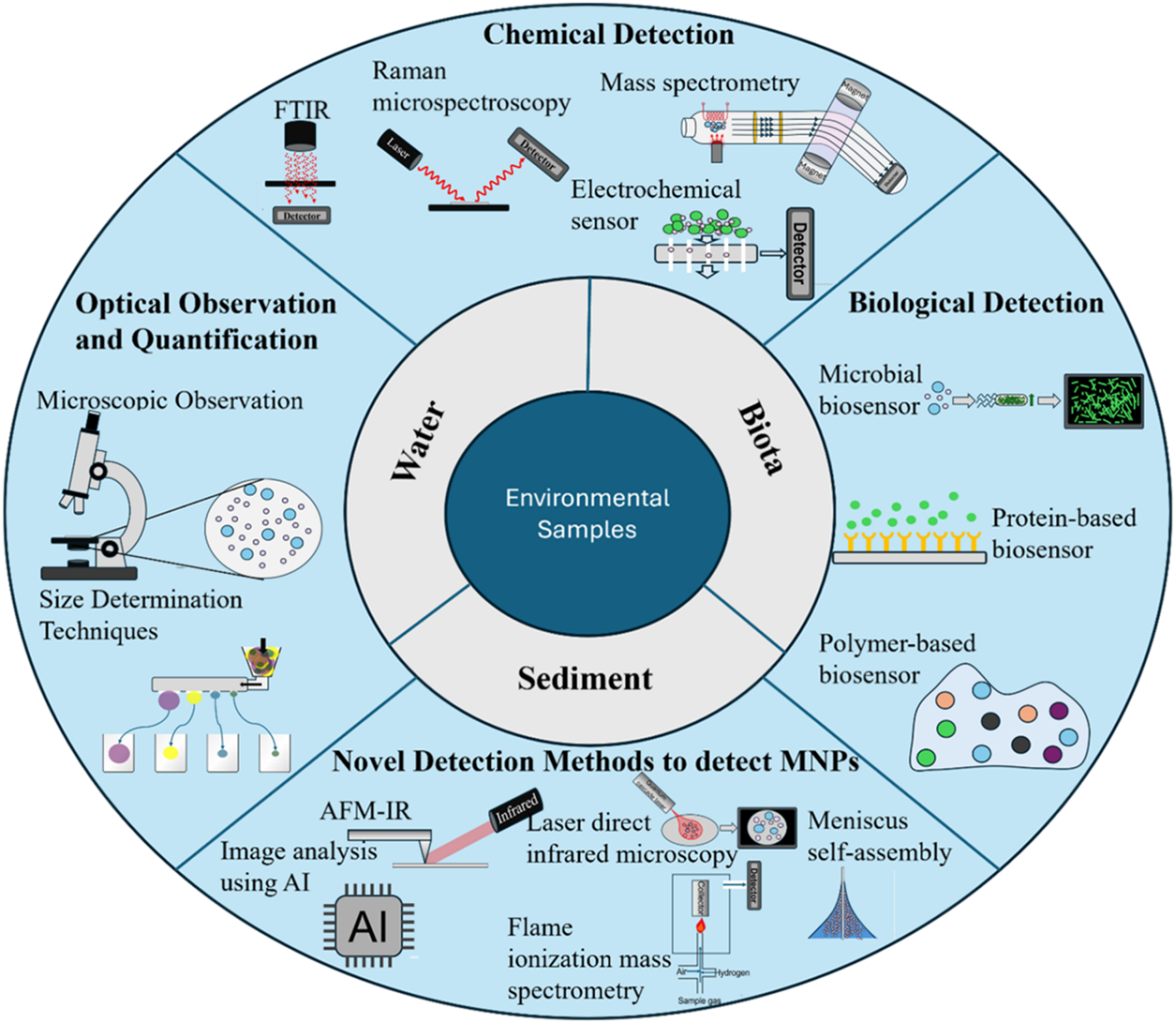
Different detection methods utilized for the identification and quantification of MNPs in complex environmental samples, and the novel detection methods to detect MNPs. There are 3 types of detection methods that can be utilized for the identification and quantification of MNPs in various complex environmental samples: optical observation and quantification to provide insights into size and morphology, chemical detection to enable quantitative and qualitative assessments of polymer types, and, lastly, biological detection for cost-effective, rapid, and in situ MNP detection. Several novel detection methods are also used to detect MNPs. Note: All elements of the figure were created by the authors.

**Figure 2:**
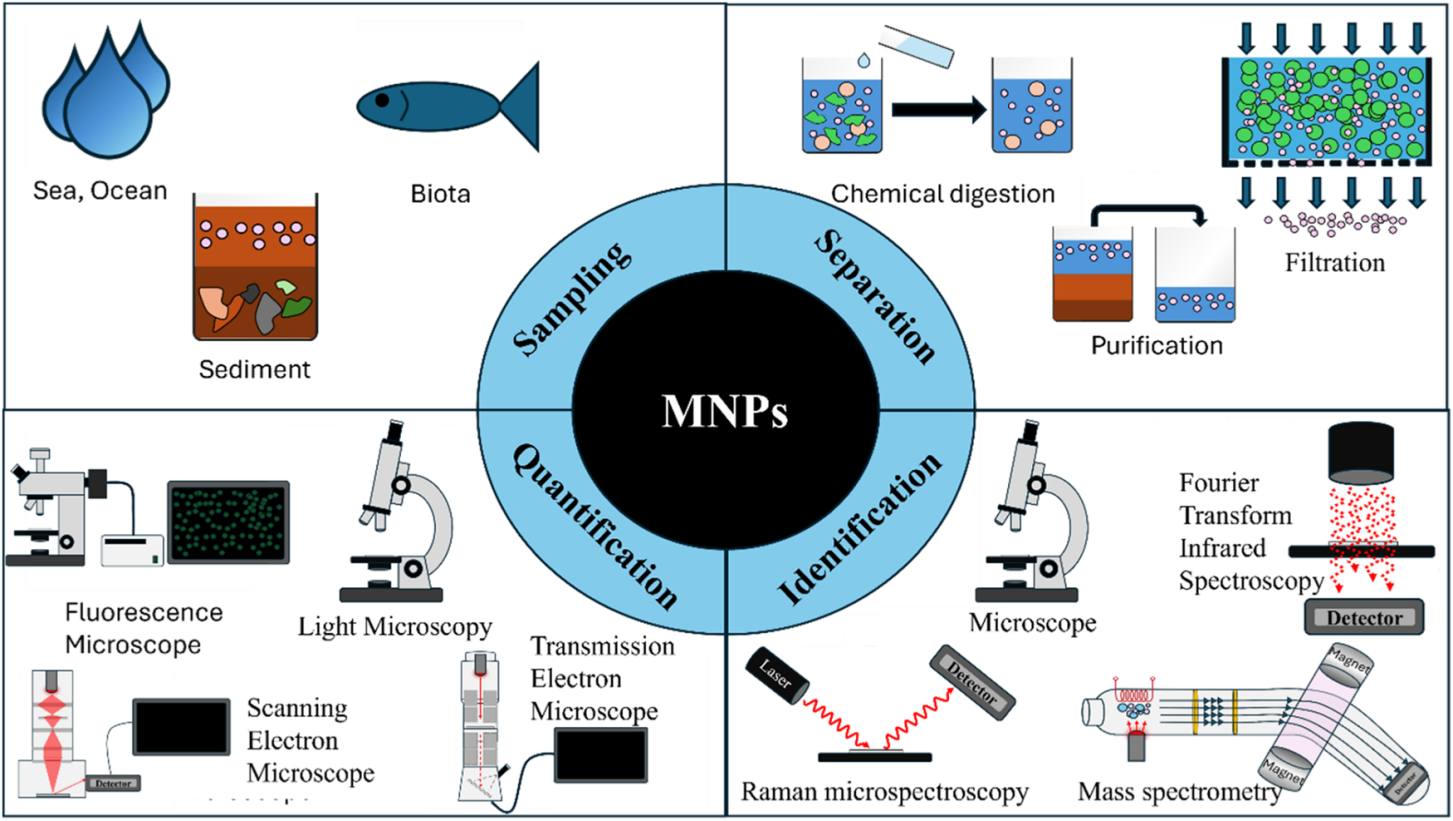
Analytical workflow of identification and quantification of MNPs, starting from sampling of MNPs, separation of MNPs from other matter, and identification and quantification of MNPs. First, sampling can be taken from the water of the sea or ocean, associated sediment, and biota. Next, collected samples can be separated from organic and inorganic matter by chemical digestion, purification, filtration, or a combination of these techniques. This is followed by identification of MNPs via microscopic observation, chemical detection, biological detection, or a combination of all three methods and quantification using microscopic techniques. Note: All elements of the figure were created by the authors.

However, there are several challenges in detecting and identifying MNPs. One such challenge is that the samples brought to the lab are not evenly dispersed throughout the environment,^10^ leading to inaccurate measurement of MNPs in the environment.^11^ In addition, sufficient concentrations are required from large volumes of samples due to the difficulty in reliably measuring MPs and NPs in low-concentration environments, leading to increased risk of sample contamination and causing misleading results.^12^ The weights and sizes of MPs and NPs may not be accurate due to their known ability to attract and deposit other contaminants on their surfaces, which changes their sizes and appearance and the characteristics of the surfaces.^13^ NPs are substantially more challenging to detect and quantify compared to MPs due to their differences in size, shape, surface-to-volume ratio, tendency to agglomerate, inherent stability, and toxicity.^14^ These differences affect their behavior in the environment, such as NP dispersal via Brownian motion, whereas MPs can sediment or float,^15^ warranting the need to research NPs separately from MPs.^16^ Moreover, researchers have a limited understanding of NP concentrations and characteristics in the environment,^17^ due to the limited tools or analytical techniques that can detect and quantify NPs accurately. This results in misleading information on the concentration of NPs in the environment^18^ and difficulty in assessing human exposure to NPs.^17^

Here, we review the current methods that are used for the identification of MNPs and discuss the novel detection methods that have been invented to improve the identification and quantification of MNPs. Although several similar reviews regarding the methods used for identification and quantification, such as the one by Adhikari and his team,^19^ we review the current advanced methods and the emerging and novel methods that further improve the identification and quantification of MNPs. The current methods can be divided into several categories including physical detection using microscopic observation, chemical detection through the compositional analysis of the MNPs, and biological detection using biosensors. We then discuss novel MNP detection methods, such as AI-based automated image analysis of MNPs and new mass spectrometry techniques, such as flame ionization mass spectrometry (FI-MS) and atomic force microscopy-infrared spectroscopy (AFM-IR). Lastly, future perspectives on MNP identification and detection that require multi-method platforms rather than single techniques are discussed.

## 2 MNP Detection Methods

### 2.1 Optical Observation and Quantification

This direct method requires the use of microscopy for visualization, size determination, identification, and quantification of MPs and NPs (Figure 3). Each microscopy method has advantages and disadvantages, depending on certain samples and research conditions (Table 1).

**Figure 3:**
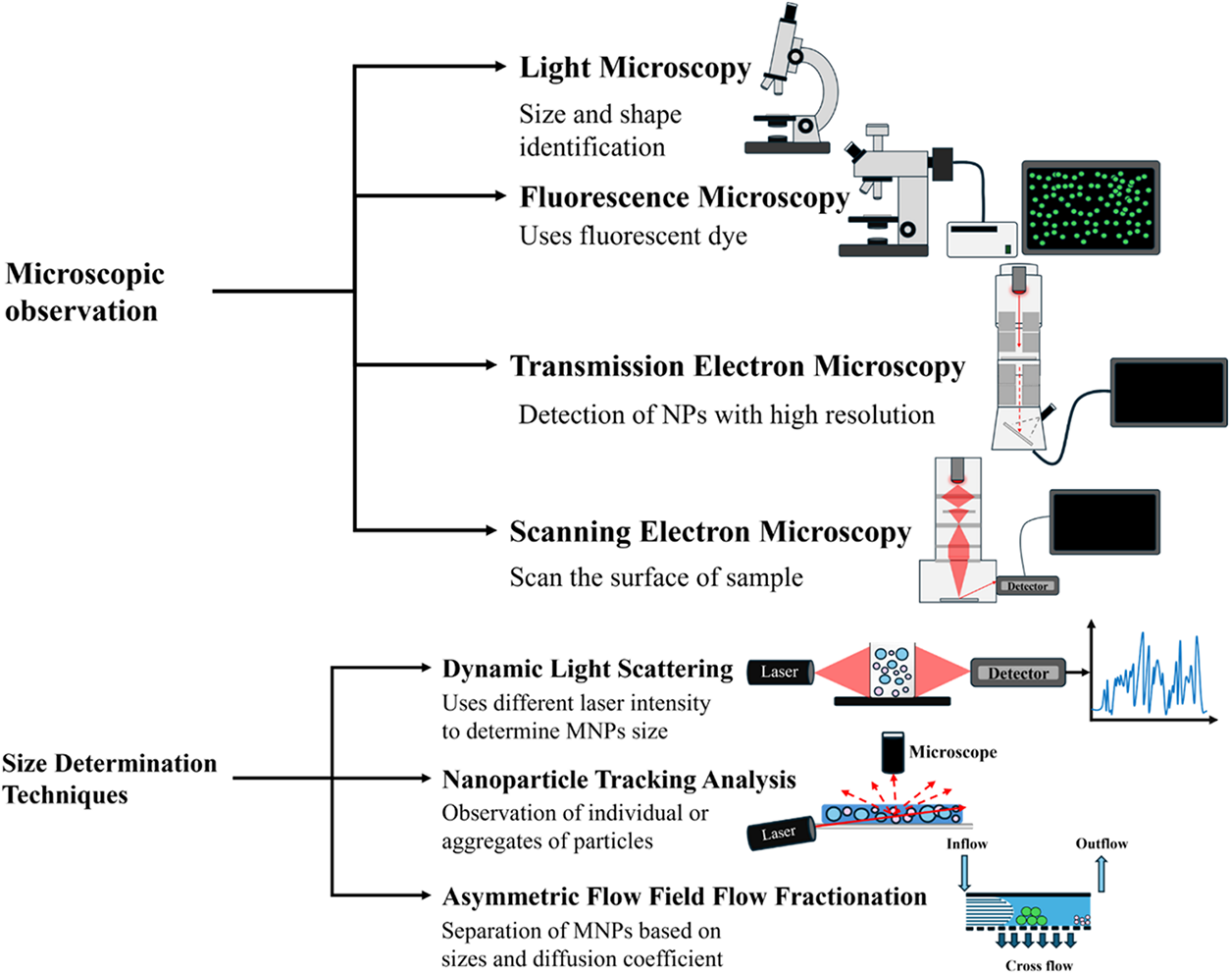
Different types of microscopes and size determination techniques used for the detection and identification of MNPs. Note: All elements of the figure were created by the authors.

**Table 1:** Different Techniques That Can Be Used Using Conventional Methods for the Detection and Quantification of MPs and NPs

**technique**	**principle**	**detection range**	**advantages**	**disadvantages**	**references**
**optical microscopy**	uses visible light to magnify images	>0.2 μm (1000× magnification)	• easy to use• cost-effective• provide real-time observation	• hard to distinguish with similar sized debris• difficult to identify MPs smaller than 200 nm	^20–22^
**fluorescence microscopy**	detects light emitted by fluorophores in samples after excitation	>200 nm	• detect transparent and white MNPs• cost-effective• fast to use	• cannot differentiate between organic and inorganic matter• chemical digestion pretreatment can damage MNPs	^23–26^
**transmission electron microscopy (TEM)**	uses a transmitted electron beam for high-resolution imaging	>1 nm	• provide morphological and structural information on MNPs• can detect NPs	• require heavy-metal staining• NPs are too similar to the cellular structure	^26–28^
**scanning electron microscopy (SEM)**	scans the sample surface with a focused electron beam	>0.5 nm (10,000× magnification)	• can identify MNPs with both organic and inorganic matter	• high maintenance costs• require skilled operators• long sample preparation time	^11,23,29,30^
**dynamic light scattering (DLS)**	measures Brownian motion via scattered light to determine hydrodynamic size	ranging from 1 nm to 10 μm	• simple to use• fast• inexpensive	• low-resolution method• only suitable for initial check• larger MPs can cover a smaller MNPs signal	^31–34^
**nanoparticle tracking analysis (NTA)**	tracks the Brownian motion of individual particles via light scattering	ranging from 30 to 1000 nm	• more precise measurement of particle size	• accidental bias from the operator	^32,35^

#### 2.1.1 Light Microscopy

Light microscopy is easy to operate, accessible, and cost-effective, with the ability to provide real-time observation of MNPs.^20^ Despite its advantages, it can be difficult to identify MNPs through light microscopy due to its similarities with other debris including shell fragments, sand, glass, and rocks with similar sizes.^21^ For the detection and identification of MNPs from water samples, large MPs with sizes larger than 1 mm were typically sorted and identified with the naked eye.^36^ For smaller MPs with sizes ranging from 500 μm to 1 mm, a light microscope can be used to aid with identification.^36^ It is possible to identify and quantify MNPs from biota samples, but appropriate pretreatment methods are required to purify and concentrate MNPs before light microscopy.^37^ To distinguish MNPs from other similar debris, the following criteria that define MNPs were established.^38^

1. No visible organic structure or cellular materials attached to MNPs.
2. If MNPs are shaped similar to fibers, they should have even thickness on all sides and three-dimensional bending of the fiber is visible.
3. MNPs are clear and uniformly colored in black, red, yellow, or blue.
4. MNPs need to be examined further under high magnification together with a fluorescence microscope if MNPs have no color to exclude any organic origin.

These criteria can be used to identify large MPs fragments with a limit of up to a size of 50 μm, but any MNPs that are smaller than 100 μm are more challenging to be identified under a light microscope,^23^ even if the light microscope can provide a maximum magnification of 1000× with a highest resolution of 200 nm.^22^

#### 2.1.2 Fluorescence Microscopy

A fluorescence microscope can be used to detect self-fluorescent or fluorescent dye-stained MNPs,^39^ with sizes greater than 200 nm.^24^ The staining techniques can be performed by either placing the MNPs on polycarbonate filter paper for direct staining with dyes or adding MNPs to a staining solution that is continuously heated and cooled to enhance the stain intensity in the MNPs.^40^ The stains that are commonly used for MNP detection are Nile Red (NR), Rhodamine B, and potentially iDye PolyPink.^41^ MPs are often detected using excitation wavelengths of 390 nm for blue fluorescence, 542 nm for red fluorescence, and 475 nm for green fluorescence after staining.^42^

Fluorescence microscopy can be used for various environmental samples including water,^25^ sediment,^43^ and biota^44^ samples. However, several pretreatments include filtration,^44^ chemical digestion, purification, and enrichment.^23^ Chemical digestion is the crucial pretreatment, as it helps mitigate the inability of fluorescence microscopy to differentiate inorganic from organic matter that can also be stained and prevents misidentification of MPs and organic matter.^26^ It is important to note that strong acidic or basic solutions used for digestion to eliminate organic debris might damage the surface of MPs, produce discoloration, and alter their size,^25^ so caution should be exercised in using harsh chemicals. Although NPs are not detectable under conventional light-based microscopy, a recent study devised a solution to detect very small MPs and NPs via NR staining.^45^ This is achieved by first aggregating the NPs and sub-MPs together into larger detectable aggregates and separating them with spiral inertial microfluidics (SIMF). Subsequently, NR was added to the NP aggregates for fluorescence microscopy. NR could stain NPs when aggregated, but no fluorescent signal was observed for nonaggregated NPs even when a higher magnification of the microscope was used,^45^ indicating that this method can be used to aggregate and observe NPs that are usually invisible under a conventional light microscope.

#### 2.1.3 Transmission Electron Microscopy

Electron microscopy (EM) provides high-resolution power to visualize and detect MNPs, due to the short wavelength of the electrons compared to light microscopy.^29^ For transmission electron microscopy (TEM), the electron beam was emitted under the MNP sample, requiring a high electron voltage of up to 300 kV and a thin sample.^27^ The TEM image can provide morphological and structural information on the interior of the MNPs, providing information regarding MNP origin, degradation, and the type of aggregation.^28^ Furthermore, TEM has a high resolution of less than 1 μm, making it suitable for the detection and identification of NPs.^27^ TEM can be employed for environmental samples, including water, sediment,^46^ and biota samples^47^ to analyze MNPs. However, extensive and complex pretreatment are required to fix a thin layer of the sample for TEM visualization.^48^ For instance, TEM showed that NPs agglomerate on soil.^49^ This is due to the amorphous structure of NPs and the weak elastic interactions with electrons requiring heavy-metal stains, which can change the chemical structure of NPs.^26^ Hence, TEM should be coupled with other tools or techniques to improve the identification and quantification of NPs.^22^

#### 2.1.4 Scanning Electron Microscopy

Similar to TEM, SEM allows the differentiation of different sizes of MNPs greater than 0.5 nm with up to 10,000× magnification.^23^ Furthermore, energy dispersive spectroscopy (EDS) can be equipped together with SEM to analyze the interaction of MNPs with heavy metals or with other inorganic matters.^50^ The major advantage of using SEM compared to an optical microscope and TEM is that SEM can identify MNPs together with both organic and inorganic matter including clay minerals, quartz, and calcite attached to the surfaces.^30^ However, the coating and additives used on the samples could be vulnerable to misidentification of the polymer types of MNPs.^11^ SEM was used in the analysis of MNPs in water-based environments, where rough and edged surfaces were observed in MNPs from the surface, coastal, and lake water,^51–54^ whereas smooth surface morphology and irregular edges were observed in MNPs from wastewater.^55^ In addition, pretreated MNPs by filtration and chemical digestion in biota samples can be identified and analyzed by SEM.^56^

Another new SEM alternative, environmental SEM (E-SEM), was developed for the environmental analysis of the MNP samples. E-SEM uses low pressure and a nitrogen atmosphere instead of high pressure used in typical SEM, resulting in the lower degradation of the sample.^23^ E-SEM also requires less preparation time and lower cost, as the sample coating is not needed, allowing the samples to be used for other analyses.^57^

#### 2.1.5 Size Determination Techniques

Dynamic light scattering (DLS) and nanoparticle tracking analysis (NTA) use light scattered from the particle to determine the size of the MPs and NPs in the sample through Brownian motion. DLS uses a laser with different intensities passing through MNPs to determine MNP sizes, which is the most common technique that can be employed.^31^ DLS offers a simple, rapid, and cost-effective assessment of particle sizes ranging from 1 nm to 10 μm at concentrations between 10^8^ and 10^12^ particles/mL.^32^ However, it is a low-resolution method that is suitable only for an initial evaluation of samples.^33^ DLS is also not ideal for complex samples, as the larger MNP signal can block the smaller MNP signal, resulting in a skewed size distribution.^34^ Nonetheless, DLS was employed for environmental samples such as the measurement of NPs in seawater, gaining reliable results by filtrating the seawater at 1.2 μm before analysis.^58^ It was also used to study the detection of NP contamination in plastic labware used in laboratory settings.^59^

On the other hand, NTA uses a microscope and a digital camera to perform observation of individual or aggregates of particles.^60^ NTA can differentiate between two particles or aggregates through diffusion, Brownian motion, or intensity of light scattering.^35^ The individual particle-by-particle analysis of NTA offers a more precise determination of particle sizes ranging from 30 to 1000 nm at concentrations of 10^7^–10^9^ particles/mL and particle size distributions for colloidal solutions containing multimodal particles.^32^ However, NTA has several crucial disadvantages. The analysis of the image captured requires direct input from the operator, so this may cause an accidental bias in the detection and size determination of the MNPs. Next, NTA cannot detect NPs that are smaller than 20 nm, but the detection limit can be increased using UV light instead of using red light sources, which are typically used in NTA.^35^

Field flow fractionation (FFF) is another powerful technique to separate complex samples from organic and inorganic materials, ions, and residues produced from sample preparation.^61^ The separation of the sample was determined by the balance between the diffusion of particles and the applied external force.^62^ When the flow was introduced into the channel, smaller particles were separated from the larger particles at different velocities, where different types of forces were applied perpendicular to the channel to accumulate the particles, which were transported to the detector.^63^ There are several types of FFF depending on the field force applied, with asymmetric flow field flow fractionation (AF4) used for separation of the NP sample from 10 nm to 100 μm.^61^ While FFF has an impermeable membrane in the channel, AF4 has a semipermeable membrane, where the flow will pass through the semipermeable membrane, creating a crossflow and allowing the particles to accumulate on the walls of the channel.^64^ The advantages that AF4 presents are that the process does not alter the sample, has high flexibility and versatility in terms of the type of crossflow used, and shows minimal sample preparation.^65^ However, AF4 cannot distinguish between differently shaped particles when they are in the same size range and between single particles or aggregates. Despite this, the disadvantages of AF4 can be addressed by coupling AF4 with detectors including DLS, NTA, multiangle light scattering (MALS), and inductively coupled plasma mass spectrometry (ICP-MS).^66^

#### 2.1.6 Integration of Microscopy into Multi-Method Platforms

The microscopes and determination size techniques mentioned above are suitable for the identification and quantification of MNPs through visualization and determination of the size of MNPs nondestructively. However, light microscopy and fluorescence microscopy face challenges when identifying and quantifying NPs. TEM and SEM are more suitable for visualizing NPs, providing more information on their morphology, degradation, and origin. These microscopes can work in tandem with size determination techniques, including DLS, NTA, and AF4, and spectroscopic techniques such as Fourier transform infrared spectroscopy or Raman microspectroscopy, for a complete analysis of the size, material type, color, and shape of MNPs collected in a certain environment.^67^

### 2.2 Chemical Detection

Chemical detection is used to understand the compositional information on MPs and NPs (Figure 4 and Table 2). Information on the chemical composition of MNPs are crucial, as it provides significant insights into the source of release, transport mechanisms, and potentially linked hazardous chemical substances.^68^

**Figure 4:**
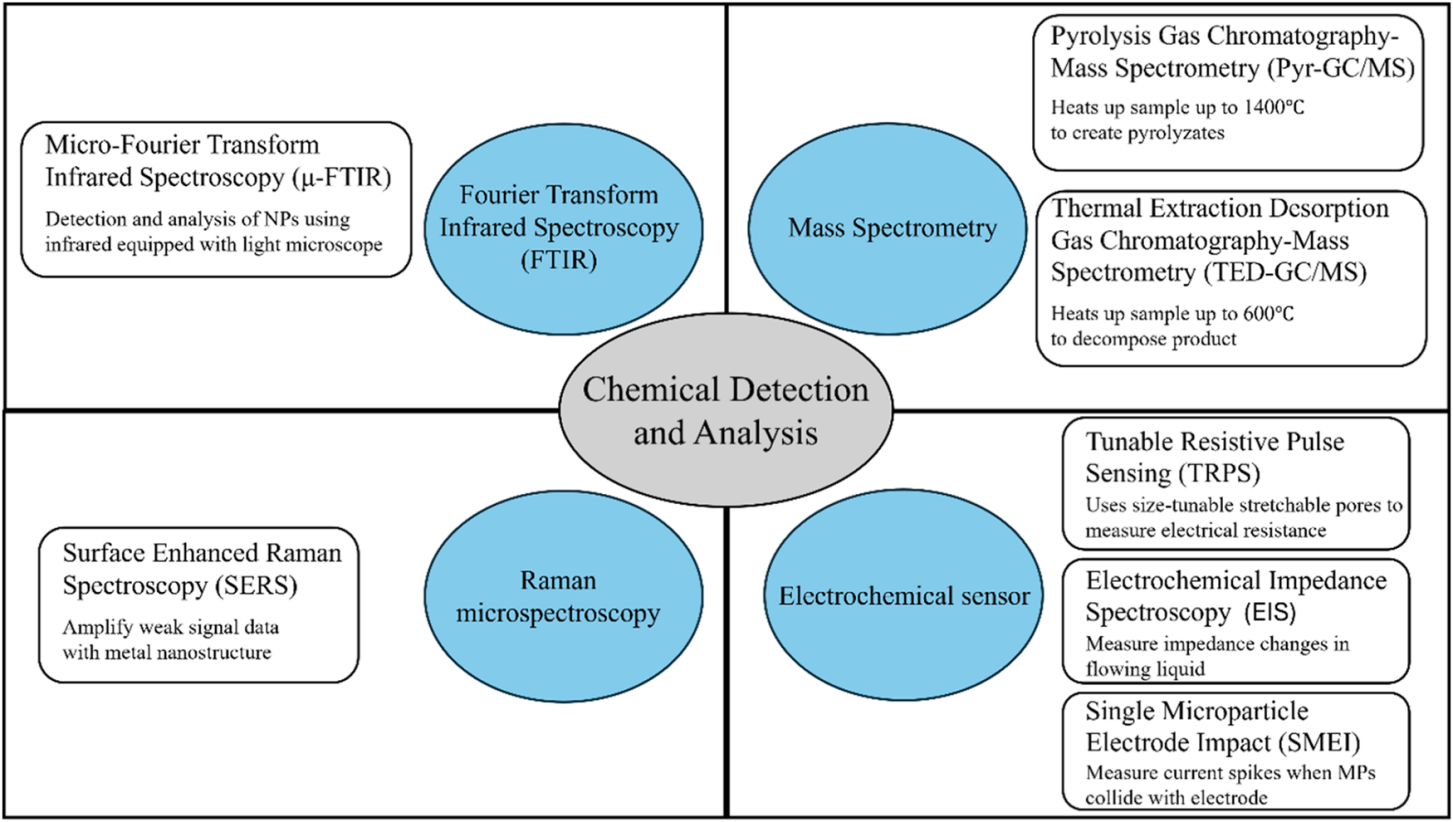
Different methods for the chemical detection and analysis of MNPs. Note: All elements of the figure were created by the authors.

**Table 2:** Techniques That Can Be Used for the Chemical Detection of MPs and NPs

**technique**	**principle**	**detection range**	**advantages**	**disadvantages**	**references**
**micro-Fourier transform infrared spectroscopy (μ-FTIR)**	irradiation with IR light causes vibrational transitions, creating a unique “fingerprint” absorbance spectrum	10 μm	• performs single-particle analysis• provides morphological and size information on MPs when coupled with a microsocpe• nondestructive	• limited accessibility• high maintenance costs• lack of skilled operators• filter is not optimal• only applicable for bulk analysis of MPs• pretreatment can be extensive• sample can be degraded	^11,69,70^
**Raman microspectroscopy**	measures inelastic scattering of light (Raman scattering) to create a vibrational spectrum	>1 μm	• provides high-resolution images• water does not interfere with the analysis	• vibrational information can be interfered with by fluorescent organic and inorganic matter• produced a very weak signal	^71–75^
**surface enhanced Raman spectroscopy (SERS)**	amplifies the weak signal from Raman microspectroscopy using a metal nanostructure on the surface	>50 nm	• amplifies signal data produced by Raman microspectroscopy	• cannot be used for environmental samples• produces high heat	^76–78^
**thermal extraction desorption-gas chromatography-mass spectrometry (TED-GC-MS)**	heats the sample up to 600 °C, adsorbs degraded products, and then thermally desorbs them for GC-MS separation and analysis	>100 nm	• requires minimal pretreatment• less time-consuming• low maintenance costs• can perform bulk analysis	• sample may be lost• TGA crucible is too small• destructive	^79–84^
**pyrolysis coupled with gas chromatography-mass spectrometry (py-GC-MS)**	decomposes the sample up to 1400 °C, separates pyrolysis products via GC, and identifies them with MS	∼20 μm	• high sensitivity• requires a tiny amount of sample	• low sensitivity• limited by the size of the pyrolyzer• destructive	^74,85,86^
**tunable resistive pulse sensing (TRPS)**	uses a size-tunable pore that is stretchable to measure electrical resistance	ranging from 40 nm to 20 μm	• pores are elastic to suit the particle size• effective recovery of the pores	• needs to be calibrated constantly	^34,87–89^
**electrochemical impedance spectroscopy (EIS)**	uses an electrode inserted in a flowing liquid to measure electrical impedance	ranging from 300 to 1000 μm	• potentially used for in situ detection• simple to use• can differentiate organic and inorganic matter	• few research studies using environmental samples	^90–93^
**single microparticle electrode impact (SMEI)**	uses an electrode to measure the current spike generated by the collision of the sample with the electrode	ranging from 1 to 10 μm	• effective in detecting MPs• inexpensive• does not require skilled operators• portable	• only identify MPs	^91,93–95^

#### 2.2.1 Fourier Transform Infrared Spectroscopy

A widely used spectroscopic technique for MP analysis is FTIR, which uses infrared light to cause molecular vibration in a material.^11^ The interferometer will then capture the infrared (IR) radiation that interact with MPs to create an interferogram as the raw signal, which will then be “Fourier-transformed” into an absorbance spectrum with peaks that can be interpreted to certain chemical bonds.^26^ FTIR spectroscopy mostly uses the attenuated total reflection (ATR) mode in which the probe only scans the surface of the sample.^96^ The absorbance of these transitions creates a specific “fingerprint spectrum” that may be used to identify the sample by comparing the spectrum with available online spectrum libraries, including the Omnic spectral library, Nicodom polymers library, Sadtler Library, and Shimadzu materials library.^29,69^ FTIR may also be used to display the age of the MPs through the oxidation on the surface via band properties.^97^

FTIR can be used to analyze environmental samples, including water,^98^ sediment,^99^ and biota^100^ samples. However, chemical digestion of the MNP sample must be conducted to remove organic matter, followed by filtration for MP enrichment before placement in an IR transparent surface.^70^ FTIR is only applicable to bulk analysis of MPs with sizes around 100 μm,^101^ so smaller MPs have to be observed using micro-FTIR (μ-FTIR) combined with optical microscopy.^102^ The focal plane array (FPA) detector is used with μ-FTIR to reduce the time taken to create chemical analysis of MPs by generating several thousand spectra within one measurement,^87^ thus reducing the analysis time from several days to only 9 h.^103^ Nonetheless, limitations include limited accessibility of the equipment, high maintenance costs, and the need for skilled operators. Moreover, chemical digestion of the MP sample may degrade the sample, resulting in inaccurate FTIR spectra after analysis.^11,69^ Another limitation is that it is difficult to use FTIR to analyze NPs due to their small sizes, unless bulk amounts of NPs are collected.

#### 2.2.2 Raman Microspectroscopy

Raman microspectroscopy is a form of Raman spectroscopy that utilizes a microscope to analyze the scattered energy for imaging, providing high resolution due to the usage of a laser beam for excitation.^104^ Raman microspectroscopy can be used to analyze MPs down to 1 μm.^71^ After the Raman spectra were generated, they can be compared with the available online Raman spectrum library to identify the chemical properties of the MNP sample. Raman microspectroscopy can be employed in multiple environmental samples, including water,^105^ sediment,^106^ wastewater,^107^ and biota^108^ samples. While water samples typically require filtration of nonsoluble substances,^109^ complex environmental samples, including sediment, wastewater, and biota samples, require extensive pretreatment, including digestion and density separation.^110^

Raman microspectroscopy has several advantages compared to IR imaging. First, Raman microspectroscopy uses a laser that has a shorter excitation wavelength compared to IR, allowing for better image resolution that provides better detection and identification of MNPs in the sample.^71^ Next, water does not interfere with the analysis of the samples, as Raman microspectroscopy operates in a near-visible spectrum with a broad frequency range and high resolution.^72^ Moreover, compared to FTIR, identification and analysis using Raman microspectroscopy are not affected by the shape and thickness of the MNP sample.^73^ However, the Raman signal is severely affected by dyes and other organic contaminants.^74^ Moreover, energy of scattering light for generating the Raman spectrum is very weak, so the intensity of the signal data can be difficult to be interpret.^75^ In addition, MPs have higher signal data compared to NPs, so the signal from MPs can mask the signal of NPs.^104^ The weak signal of NPs can be mitigated by doing effective sample preparation to reduce any background signal and enhancing the signal using enhancement techniques such as analyzing hundred-to-thousand spectra or through an advanced method, including surface enhanced Raman spectroscopy (SERS).^15^

Another recent advanced strategy is SERS, which can amplify the weak signal data produced by Raman microspectroscopy by using a nanostructure containing metal as the surface. The laser beam charges the nanostructure surface, which creates a localized surface plasmon resonance field, so a large, amplified Raman signal can be detected when NPs are in close contact with the field.^76^ The size, shape, properties, and distance between the nanoparticles of the nanostructure can affect the intensity of the LSPR field.^111^ Nonetheless, several issues need to be addressed for SERS. Existing studies employing SERS utilize polymeric NPs in experimental settings instead of environmental NPs because of the various shapes and sizes of environmental NPs and the potential presence of signal-interfering fluorescent contaminants.^77^ Another limitation is the excitation of the laser beam, which could increase the temperature of the nanostructure and cause plasmonic heating, resulting in degradation of the MNPs.^78^ Hence, SERS should be further optimized for the analysis of environmental MNPs.

#### 2.2.3 Mass Spectrometry

MNPs can be analyzed through their mass fraction compared to the particle count using mass spectrometry, including thermal extraction desorption-gas chromatography-mass spectrometry (TED-GC-MS) and pyrolysis gas chromatography-mass spectrometry (Py-GC-MS).

TED-GC-MS allows rapid identification and quantification of MNPs.^79^ The sample underwent TGA-based thermal desorption under nitrogen, followed by staged heating, GC separation, and mass spectrometric analysis to identify thermally decomposed compounds.^80^ The advantages of TED-GC-MS are that the sample requires minimal purification and low maintenance costs because larger compounds in the sample are not desorbed, so they do not obstruct the columns in gas chromatography.^81^ TED-GC-MS can also be utilized for bulk qualitative analysis of up to 100 mg.^74^ It is also useful for the analysis of environmental samples, where it was used to detect MNPs made of polystyrene (PS), polyethylene terephthalate (PET), and polyvinyl chloride (PVC) in snow or aerosols.^82,83^ Recently, TED-GC-MS was able to identify MNPs in a biota sample by turning the sample into powder for analysis.^83^ The disadvantage of TED-GC-Ms is that it can only accommodate a small amount of samples, so the sample may be lost or retained during transfer.^84^

The second method using mass spectrometry is py-GC-MS.^74^ Py-GC-MS decomposes samples at high temperatures ranging from 550 to 1400*°*C in a controlled atmosphere, creating pyrolyzates for mass spectrometry analysis.^85^ While Py-GC-MS can detect MNPs with sizes around 20 μm,^85^ it can detect tiny amounts of NPs (∼50 μg) with higher sensitivity than TED-GC-MS. Py-GC-MS has been used for environmental samples, including water, sediment, and biota samples.^112^ The samples were pretreated to purify and concentrate MNPs in the sample, with biota samples requiring more pretreatment by enzymatic digestion of organic matter and filtration to reduce background contamination.^112^ However, Py-GC-MS also has its own limitations. First, samples that may be injected are limited by the size of the pyrolyzer.^74^ Due to the destructive nature of Py-GC-MS, the samples could only be analyzed for a single time. Moreover, because the size, shape, density, and color of the MNPs could not be characterized by spectrometry techniques, complete MNP analysis requires other techniques, such as light microscopy.^85^

#### 2.2.4 Electrochemical Sensor

When it comes to identifying MNPs during testing, electrochemical sensing provides a number of advantages, such as mobility, affordability, and quick analysis. Tunable resistive pulse sensing (TRPS), electrochemical impedance spectroscopy (EIS), and single microparticle electrode impact have been shown to have high efficacy in identifying MNPs.^113^

First, TRPS can be utilized for single-particle analysis of MNPs with the help of microfluidics to detect the size and shape of MNPs.^88^ It uses a size-tunable pore to measure resistive pulse sensing.^89^ To achieve the necessary pore sizes, TRPS uses an elastic membrane that can be stretched.^114^ When each MNP travel through the pore, there is an increase in electrical resistance that can be used to calculate the size of the MNP traveled through the pore.^89^ TRPS can obtain the size and concentration of MNPs with sizes ranging from 40 nm to 20 μm.^34^ It is advantageous to use TRPS because the elastic pore can be tuned to suit the size of the particle and stretched without any damage, thus allowing effective recovery of the pore.^114^ The limitation is that TPRS needs to be precalibrated to obtain absolute accurate analysis of the MNPs, as direct measurements are difficult due to the constant change of the pore size.^89^ More research is required to determine if TRPS is suitable for environmental samples, as debris with similar sizes could result in false positive results.

Next, EIS uses electrodes inserted within a microchannel to detect, quantify, and analyze liquid-suspended MNPs with sizes ranging from 300 to 1000 μm.^90^ The electrodes will measure the impedance change when MNPs pass through, so the data obtained can be used to assess the electrical properties of MNPs.^91^ MNPs can be distinguished from other organic and inorganic matter based on the impedance changes, so they can be used for in situ detection.^92^ However, sterilized water, instead of water collected from the environment, was used to suspend MNPs in these studies, so more research is needed to determine if environmental water could interfere with the identification and detection of MNPs.^93^

Lastly, single microparticle electrode impact (SMEI) uses an electrode that changes current response when MPs collide with the electrode.^94^ The current response will create current spikes that can be analyzed based on the shape and frequency to generate information on the size and quantification of MPs,^93^ with a size range of 1–10 μm.^95^ It is cost-effective, portable, and easy to operate.^115^ While it is only used for MP analysis and quantification, it may be possible to identify NPs due to the similarities in the properties between MPs and NPs.^91^ The research of SMEI for the detection and quantification of MNPs is still in its infancy, but may present a promising technique for MNP detection in environmental samples.

#### 2.2.5 Integration of Chemical Detection Methods into Multi-Method Platforms

The analytical methods for identifying MNPs using the methods mentioned in chemical detection address the difficulties in characterizing different types of MNPs in environmental samples. These chemical detection methods can detect MNPs more effectively and provide better chemical information compared with light microscopy. However, chemical detection methods have limitations specific to each technique. For FTIR and Raman microspectroscopy, there remains the need for extensive pretreatment of the samples, with the potential to degrade MNPs. TED-GC-MS and Py-GC/MS are destructive to the samples and require a large amount of samples for accurate analysis. To increase their accuracy in detecting and quantifying MNPs, FTIR can be used together with Py-GC/MS to first detect the MNPs using FTIR, followed by the determination of the polymer types and mass via Py-GC/MS.^116^ Chemical detection methods can also be combined with microscopy detection methods or size determination techniques to provide morphological information and prevent degradation of the sample through chemical digestion and bulk amounts of the sample through DLS/NTA or AF4.^116^

### 2.3 Biological Detection

Biosensors are emerging sensing methods for MNPs. A biosensor usually consists of three main parts, including a bioreceptor, transducer and a detection device.^117^ Various bioreceptors that can be utilized for MNP detection are discussed (Figure 5 and Table 3). Compared to traditional physicochemical methods explained in previous sections, biosensors provide several advantages such as high sensitivity, low detection limit, low cost, and rapid response time.^118^

**Figure 5:**
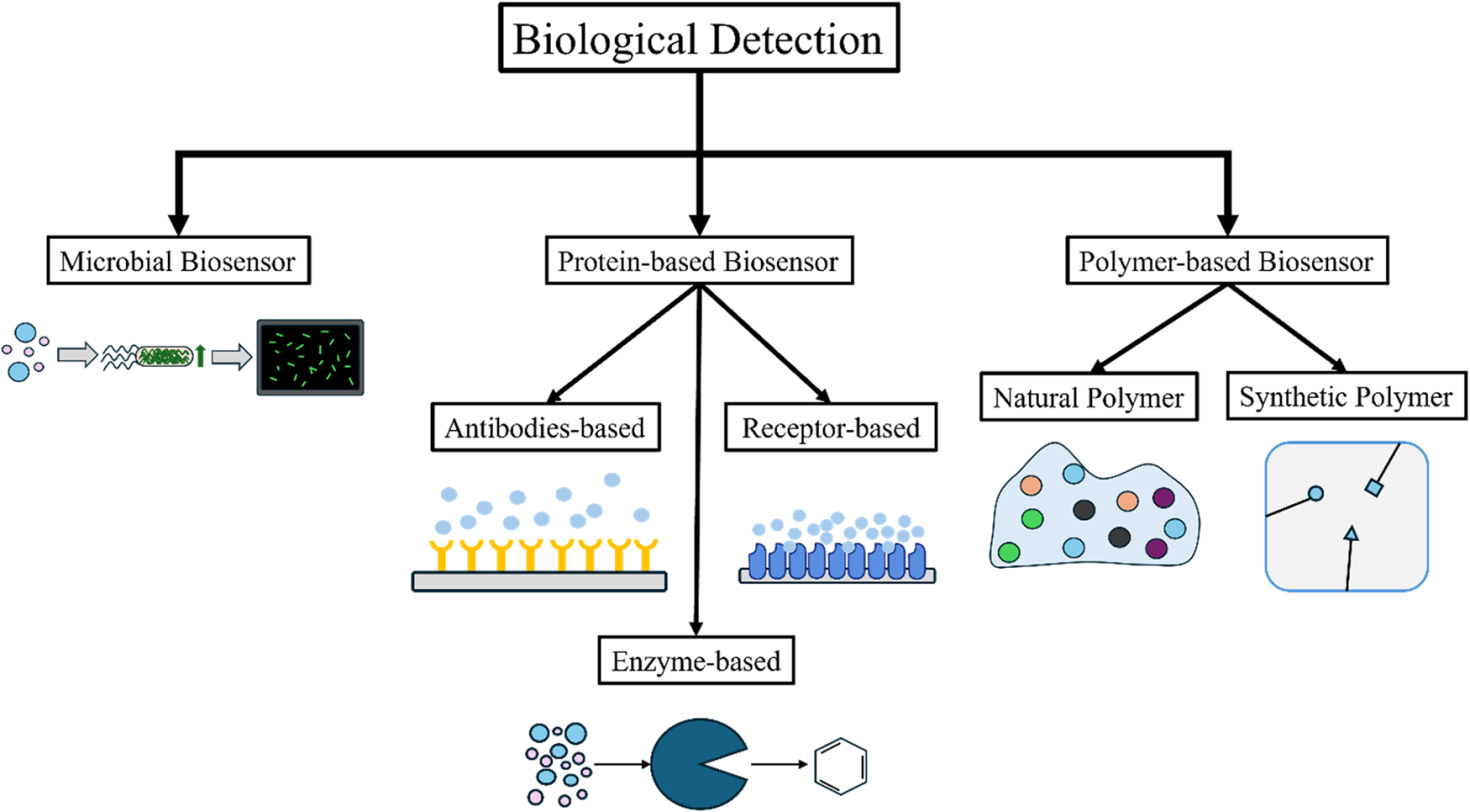
Three types of biosensors used for the biological detection of MNPs include microbial, protein-based, and polymer-based biosensors. A microbial biosensor uses live bacteria with a fluorescent reporter system to bind with MNPs to produce a fluorescent signal. A protein-based biosensor detects MNPs by binding with MNPs, triggering a reaction. A polymer-based biosensor traps MNPs and can detect MNPs by measuring the impedance level of the polymer. Note: All elements of the figure were created by the authors.

**Table 3:** Techniques That Can Be Deployed Using Biological Methods for the Detection and Identification of MPs and NPs

**technique**	**principle**	**detection range**	**advantages**	**disadvantages**	**references**
**microbial biosensors**	adding modified live bacteria to the MNP sample to generate a fluorescent signal	1 ng/mL to 1 mg/mL	• very high sensitivity• inexpensive• very low detection limit• easy to use• minimal pretreatment required	• limited bacteria can be utilized• only specific MNPs can be detected• not portable	^119–123^
**antibody-based biosensors**	binds MNPs to immobilized antibodies on the surface	24 nm	• high binding affinities with MNPs• high specificity• can detect specific polymers in environmental samples	• only specific polymers can be detected• data from ELISA requires standardization	^119,124,125^
**enzyme-based biosensors**	binding MNPs to the enzyme to trigger a reaction on the substrate	0.02 μM	• required a low amount of the sample• rapid response time	• limited enzymes can be utilized• limited research conducted• only specific polymers can be detected	^119,126,127^
**receptor-based biosensor**	uses a probe tip to scan the surface of the sample	1 ng/mL	• required a low amount of the sample• rapid response time• can be used for an environmental sample	• limited receptors can be utilized• only specific polymers can be detected	^128,129^
**polymer-based biosensors**	polymer binding to the surface can create strong binding to MNPs	ranging from 0.1 μm to 1 mm	• has a very low detection limit of 10^–11^ to 10^–5^ M	• limited research conducted• no synthetic polymer has been developed	^119,130^

#### 2.3.1 Microbial Biosensor

Cell-based biosensors have been developed and employed for the detection of numerous targets including biotoxins, biomarkers, and environmental pollutants.^131^ Microbial biosensors are employed by adding live bacteria to the MNP sample. A biochemical reaction will be activated or deactivated when bacteria bind to MNPs, which will cause changes in the signal produced by the transducers, which can be either optical or electrochemical.^119^ For optical transducers, bacteria are genetically engineered to have a fluorescent reporter system to allow visualization of the bacteria.^132^ Moreover, bacteria possessed the ability to aggregate MPs from large sample sizes, for convenient recovery and subsequent detection or analysis.^133,134^

A recent study employed*Pseudomonas aeruginosa* PAO1/p_*cdrA*_-*gfp* as a live biosensor that forms biofilms on MPs and synthesizes GFP, for subsequent quantification of the GFP fluorescence level via confocal microscopy.^120^ Results showed a positive correlation of the GFP fluorescence level produced by the biosensor with MP concentration in the samples. As this biosensor can detect low MP concentrations of around 1 μg/mL or higher rapidly, it possesses the potential to be deployed in the field for in situ detection.^120^

Other than using GFP-based systems, bioluminescence is an alternative for rapid and sensitive detection of MPS, where a similar study employed recombinant *Escherichia coli* containing *Photinus pyralis* firefly luciferase (lucFF) to detect acrylic acid in plastics.^135^ A microplate reader is needed to measure the bioluminescence of this biosensor.

Another strategy was the microbial detection of the degraded products of plastics. *Comamonas thiooxidans* strain S23 was developed as a microbial-based biosensor to detect PET breakdown products within hours.^121^ It can utilize a tripartite tricarboxylate transporter (TTT) and mono- and dioxygenases encoded in the *tph* operon to break down PET to TPA and use TPA as an energy source, so insertion of a superfolder GFP (sfGFP) gene fused to the *tphC* promoter confers the bacterium the ability to detect TPA with a low detection limit of 10 μM. Its mutant strain with the deletion mutation in the *thphA2-A1* gene could even detect TPA at a lower detection limit of 1 nM.^121^ However, it is only applicable to PET breakdown products including TPA, MHET, and bis­(2-hydroxyethyl) terephthalate (BHET). A similar study also modified the transcription factor XylS from *Pseudomonas putida* to recognize and bind to TPA and phthalic acid (PA).^122^

Despite their listed advantages, microbial biosensors still require heavy instrumentation including a fluorescence/confocal microscope and a microplate reader to accurately measure the fluorescence level produced by the microbes. Hence, a portable detection device that allows in situ detection of MNPs is needed.^119^ Furthermore, there are few microbes discovered to express the receptors or signaling molecules for binding specific MNPs, rendering it challenging to detect and quantify specific MNPs in environmental samples.^136^

#### 2.3.2 Protein-Based Detectors

Proteins used for biosensing include antibodies, enzymes, receptors, and short peptides. First, antibody-based biosensors are most commonly used in MNP detection due to their high binding affinities and high specificity, but this high specificity indicates that they cannot be used for the detection of a wide range of MNPs.^119^ Several antibodies can be utilized for an antibody-based biosensor. Detection of bisphenol A (BPA) in epoxy resin and polycarbonate plastics was achieved by covalently binding pAbs on a gold–quartz crystal coated with a carboxylated polyvinyl chloride polymer membrane.^124^ When BPA binds with pAbs, the binding creates a potentiometric reaction to quantify the BPA concentration. A recent development was able to create polystyrene (PS)-specific antibodies for the detection of PS at a size of 24 nm.^125^ PS was conjugated with a carrier protein to be used for rabbit immunization to create PS-specific rabbit pAbs. By using the enzyme-linked immunosorbent assay (ELISA), PS-specific antibodies could detect and analyze PS particles in different environmental samples. This study showed the possibilities of developing reliable and efficient antibody-based biosensors for MNPs analysis. However, the data generated by ELISA from antibody-based biosensors needs careful standardization and calibration when analyzing environmental samples.^125^

Enzyme can be used as well to create enzyme-based biosensors by binding to the target sample and trigger a reaction on the substrate.^119^ An approach that can be used is to study the reaction of different types of enzymes to certain MNPs. Only tyrosinase was able to detect the presence of BPA by using an amperometric sensor, where it showed an extremely low detection limit of 0.02 μM with 5 min of response time.^126^ Another enzyme that can be utilized for an enzyme-based biosensor was the laccase enzyme.^127^ A study conducted by Rivera-Rivera et al. uses surface plasmonic resonance (SPR) with the laccase enzyme as the receptor to create a biosensor.^127^ The laccase enzyme was able to provide high sensitivity and was capable of detecting PE, poly­(methyl methacrylate) (PMMA), and PS with detection limits of 7.5 *×* 10^–4^ μg/mL, 3.7 μg/mL, and 68.3 μg/mL, respectively. In addition, SPR with a laccase enzyme biosensor does not require any pretreatment of the samples, which is suitable for environmental samples.^127^

A receptor-based biosensor is similar to an antibody-based biosensor in which receptors can bind to MNPs, so it is highly effective for biosensing too. A recent study developed a human estrogen receptor α protein combined with an SPR sensor.^128^ The human estrogen receptor α protein was immobilized by plasma to the metal surface of the SPR, so it could bind to and detect PS, PVC, and PE. Another study utilized a plasmonic gold nanosurface with an estrogen receptor for the detection of NPs in seawater,^129^ where it required a low amount of samples (2 μL) with only 3 min of response time and a low detection limit of 1 ng/mL.

For the analysis of environmental samples, these protein-based biosensors still face several challenges, including stability, interference with detection, and costs. Moreover, proteins are susceptible to environmental factors, leading to protein degradation over time.^136^ Despite protein-based biosensors having high specificity, environmental samples contain various substances that can interfere with MNP detection.^137^ Furthermore, MNPs found in the environment vastly vary in structure, which raises the need for a universal receptor that can detect degraded MNPs.^136^ Moreover, extracting the proteins used for these biosensors requires downstream processing and purification, adding more to the overall costs of upscaling.^137^

#### 2.3.3 Polymer-Based Biosensor

While uncommon, polymers can be used to detect MNPs, as they have high electrostatic attraction, thus generating strong binding to MNPs.^119^ An example of the polymer-based biosensor was using extracellular polymeric substances (EPS) extracted from the cyanobacterium *Gloecapsa gelatinosa* to detect MPs.^130^ Extracted EPS were then immobilized on a gold surface by spin coating. For MP detection, EIS was used to measure the impedance level of the EPS that bind to the MPs,^119^ so MPs trapped in the EPS will lower the resistance. This biosensor was able to detect MPs with sizes ranging from 0.1 μm to 1 mm with a very low detection limit of 10^–11^ to 10^–5^ M.^130^ However, it has challenges in selectivity for MNPs in environmental samples, due to the presence of other pollutants that interfere with MNP detection.^130^

#### 2.3.4 Integration of Biological Detection into Multi-Method Platforms

The emergence of biological detection using biosensors allows for higher sensitivity in detecting MNPs due to their high binding affinities with MNPs. Under the current progress in biosensors in detecting MNPs, these biosensors may work in combination with TED-GC-MS or Py-GC/MS to validate the specific polymer type that was detected by biosensors. In addition, biosensors can be used together with fluorescence microscopy for the identification and quantification of MNPs that bind to the biosensors.

### 2.4 Novel Detection Methods to Detect MNPs

To further increase the sensitivity and the accuracy of MP and NP detection, several novel detection methods, such as artificial intelligence (AI)-based automated image analysis, atomic force microscopy (AFM), and flame ionization mass spectrometry (FI-MS), have been developed and combined together with other available detection methods in recent years.

#### 2.4.1 Automated Image Analysis Using AI

AI has advanced over the years, becoming more accurate and faster for environmental studies.^138^ AI can be used to automate the identification and quantification of MPs and NPs, which improves accuracy and efficiency for MNP identification.^139^ To analyze photos using Bruker’s OPUS© software, a unique analytical pipeline was developed utilizing Python and Simple ITK image processing modules. Data sets from focal plane array (FPA) mFTIR mapping of samples with up to 1.8 million single spectra may be analyzed. An image analysis system that gave details on the sizes and quantities of each MP and NP found was produced as a result of this innovative technique.^140^ Furthermore, this technique significantly improves the data quality and shortens the time required to analyze challenging FTIR imaging data. A recent study using an AI-assisted nanodigital in-line holographic microscope (AI-assisted nano-DIHM) for the detection and characterization of NPs showed that it could perform a particle characterization rate of around 1.4 objects per second and a classification rate of 25 objects per second.^141^

However, there are several limitations in using AI for image analysis of MNPs, which include the need for the creation of a data set with accurate identification, readiness, interpretability, transparency, and reproducibility of AI. To be able to accurately detect and analyze MNPs in a sample, a labeled and accurate data set is required to train the AI. If not, an unlabeled, low-quality data set could lead to misclassification of MNPs.^142^ Furthermore, current AI models are limited to controlled or laboratory settings because environmental samples could pose sample complexity issues.^143^ This raises concerns about the reproducibility of MNP studies involving AI models, as there is a lack of detailed methodology in how the AI models are trained, which poses a challenge for the cross-validation of the results.^143^

#### 2.4.2 AFM-IR

Atomic force microscopy (AFM) is recently utilized to enhance identification and characterization of NPs.^14^ AFM works by scanning the surface of the sample using a sharp probe tip by applying constant force using a piezoelectric mechanism, so that the force between the probe tip and the sample is measured to generate high-resolution images.^26^ AFM can provide precise information on the surface roughness, degradation rate, and the interaction of MNPs with organic or inorganic matter, making it a suitable instrument for environmental studies of MNPs.^20^ The key advantages of AFM include the ability to detect extremely small NPs (∼0.3 nm),^23^ simple sample pretreatment,^144^ and generation of 3D images.^20^ However, AFM still presents some limitations. First, the samples can be easily contaminated by non-MNP samples during AFM scanning.^145^ Next, the probe tip might damage the sample during scanning, leading to inaccurate imaging.^146^ Moreover, AFM is tedious and slow to scan across the sample, so preconcentration of samples by ultrafiltration is needed.^147^

Since AFM cannot identify the different types of plastics present in the sample, it can be utilized with other methods for the chemical analysis of the sample^148^ such as AFM-IR and tip-enhanced Raman spectroscopy (TERS). The AFM-IR can record spectral and special information of NPs in the range of 50 nm,^29^ where AFM will scan the surface sample and generate an image first, followed by IR to analyze the thermal and mechanical properties of the samples.^11^ This is achieved by the AFM probe tip that senses the surface of the sample when the IR laser was absorbed by the sample, which was picked up by the cantilever.^149^ Further combining AFM-IR together with Pyr-GC/MS can help to identify PE and PVC NPs with sizes ranging from 20 to 1000 nm from the drinking water treatment plant.^150^

AFM can also be paired with Raman microspectroscopy for MNP detection to enhance the sensitivity and resolution of Raman microspectroscopy.^29^ The tip-enhanced Raman spectroscopy (TERS) uses a probe with a tip to scan the surface of the sample with Raman spectroscopy, thus enabling a high resolution of around 10–20 nm.^151^ However, the material, shape, and size of the probe tip affect the sensitivity and resolution of TERS, so specialized skills are needed to operate TERS.^152^ Given the slow speed of TERS, it can only scan localized and near field part of the surface sample, but not the entire sample.^151^

#### 2.4.3 Flame Ionization Mass Spectrometry

Recently, FI-MS has been developed for rapid MP and NP detection and quantification.^153^ A dry sample, such as powder, dirt, or tissue, is burned or heated in front of the MS intake in FI-MS. Polymers like PET and PS may be broken down and ionized by FI-MS, enabling analysis in 10 s per sample. Additionally, FI-MS requires smaller samples and offers great specificity because of the comprehensive molecular information it delivers. Hence, FI-MS could detect PET, PE, and PVC. For PVC MPs, FI-MS was able to detect three aromatics including naphthalene, 1-methylnaphthalene, and phenanthrene. For PE MPs, three hydrocarbons were detected including hepta-1,6-diene, octa-1,7-diene, and nano-1,8-diene. For biological and environmental applications, FI-MS was capable of detecting MPs in soil and NPs in animal tissues. However, FI-MS requires skilled operators to burn the sample before the MS intake, so its application is currently limited.^153^

#### 2.4.4 Laser Direct Infrared (LDIR) Microscopy

The detection and quantification of MPs from various environments including oceans, soil, and biological tissues have been further improved by LDIR imaging.^154^ By using a tunable quantum cascade laser (QCL) as its IR source, LDIR can selectively target MPs, ignoring the empty spaces.^155^ When compared to both Raman and FTIR, LDIR can provide faster analysis and more precise wavelength–frequency, requiring only 1 s per particle for the analysis of MPs.^156^ Coupled with a high-magnification optical camera, LDIR is capable of detecting MP sizes of around 10 μm.^154^ Compared to μ-FTIR or Raman microspectroscopy, which analyzes the entire sample, including the empty spaces, LDIR initially scans the entire sample to identify MPs before imaging, substantially reducing the time for analysis. The downside of using LDIR is that it is unable to distinguish between two particles if they are close to each other, and they are recorded as one particle.^157^ Another downside is that LDIR uses a narrower infrared band compared to μFTIR, leading to a higher tendency to misidentify weathered MPs.^158^ Despite this, the rapid analysis and efficient processing of a large sample area make up for its downsides.^154^

#### 2.4.5 Meniscus Self-Assembly

Meniscus self-assembly is an NP-collection method that relies on the evaporation of solvent to align NPs uniformly at the solid–liquid interface on the surface.^159^ The sample is first filtered through a filter membrane, and the filtered NPs are concentrated using the meniscus self-assembly and observed using fluorescence microscopy or SEM.^159^ The method facilitates in concentrating NPs uniformly on a surface with sizes less than 1 μm.^159^ Consequently, this approach is relatively easy to use and cost-effective with high efficiency.^160^ However, the accuracy of this method is heavily influenced by the concentration and particle size. Furthermore, larger MNPs can be displaced due to their weight and large size, so this method is more suitable for NPs.^159^ Meniscus self-assembly is still at an early stage due to the lack of analysis using environmental samples, so a real-world application is required.^159^

## 3 Future Perspectives

The future of MNP sensing is centered on standardization, automation, and the development of innovative, extremely sensitive techniques capable of overcoming the major limitations of the present approaches. The fundamental problem is the ″analysis bottleneck″ created by the enormous diversity of particle size, shape, polymer type, and chemical content, all of which exist within complex environmental and biological matrices.^11^ Hence, there should be an initiative to develop certified reference materials (CRMs) for MNPs to standardize the data obtained.^161^ Our future vision calls for integrated, multi-method platforms that offer full data (size, mass, count, polymer identification, and even surface chemistry), rather than depending on a single technique. This could be achieved by combining optical methods to observe MNP morphology and determine MNP sizes, followed by chemical methods, such as μ-FTIR or Raman microspectroscopy, to analyze MNP material type.

Next, the current methods for visual counting and manual manipulation of samples are time-consuming and error-prone, so it is possible to integrate machine learning and AI. AI algorithms may be trained to detect and identify MNPs quickly when coupled with the detection techniques for MNPs, thus enhancing the MNP analysis speed, throughput, and accuracy. Hyperspectral imaging and automation are alternatives that can combine chemical detection techniques with automated stages and software for quick scanning of whole filters, resulting in massive data sets for analysis by machine learning (ML).^117,162^

Another challenge is the detection and characterization of extremely small NPs, resulting in fewer studies of NPs than MPs. Current isolation and detection approaches risk losing or contaminating the NP samples. Hence, effective and standardized methods for collecting NPs from vast quantities of water, such as ultrafiltration or continuous flow centrifugation, and digesting organic materials without damaging NPs are urgently required.^11^ In addition, there is a serious absence of well-defined NP standards with established sizes, shapes, and polymer composition. The development of certified reference materials is critical for validating novel analytical techniques and calibrating equipment, thereby facilitating interlaboratory comparisons.^161^ Similarly, new technologies must also be extensively compared to existing techniques and sample matrices.

In conclusion, many techniques are developed for MNP detection, quantification, and characterization, each with its own advantages and limitations. The future of MNP analysis does not fall on a single approach but rather an integrated, multi-method workflow that combines complementary techniques to offer full data on particle size, count, mass, and polymer identity.
